# Persistence and recurrence after removal of idiopathic epiretinal membrane

**DOI:** 10.1038/s41433-024-03429-y

**Published:** 2024-10-25

**Authors:** Felix F. Reichel, Eduardo Labbe, Faik Gelisken, Immanuel P. Seitz, Sherif Hagazy, Spyridon Dimopoulos

**Affiliations:** 1 University Eye Hospital, Centre for Ophthalmology, University Hospital Tübingen, Tübingen, Germany; 2 Clinica Alemana-Universidad del Desarrollo, Santiago, Chile; 3 Research Institute of Ophthalmology, Cairo, Egypt

**Keywords:** Outcomes research, Risk factors

## Abstract

**Objectives:**

To analyse the incidence of persistence and recurrence after the peeling of idiopathic epiretinal membrane (ERM) and to describe its clinical features.

**Methods:**

This retrospective study included 666 eyes (645 patients) that underwent macular surgery for ERM removal. Optical coherence tomographic (OCT) images taken within three months after surgery and at the following visits, clinical parameters and surgery related factors were analysed to investigate the incidence and associated factors of ERM persistence and recurrence. Postoperative ERM types were categorised depending on the size ( < 100 µm, ≥100 µm) and the location (foveal, parafoveal, outside the parafovea)

**Results:**

The mean follow-up time was 29.4 months. ERM persistence (examination within 3 months) was found in 29.6% of all eyes. Only 1.9% of the eyes presented foveal ERM persistence. Foveal recurrence, defined as reappearance or growth of persistent ERM covering the fovea, was found in 8.2%. In 84.4% of eyes with foveal ERM recurrence, postoperative persistence of ERM of varying severity were identified. None of the pre-operative or surgery related factors were found significantly associated with ERM recurrence. Persistent ERM within the parafovea was the most significant risk factor for foveal ERM recurrence.

**Conclusion:**

Recurrence of ERM is generally preceded by the persistence of ERM fragments found in the early postoperative period. Growth of ERM persistence from the parafoveal region was often the origin of foveal ERM recurrence. Insufficient peeling seems to be the most significant predisposing factor for foveal ERM recurrence.

## Background

An epiretinal membrane (ERM) is a semi-translucent, fibrocellular tissue on the surface of the inner limiting membrane (ILM) that can lead to decline in visual acuity and metamorphopsia [1]. Idiopathic ERM, often associated with posterior vitreous detachment, is the most common form and its prevalence increases with age. Early large population studies based on the clinical examination assessed a prevalence of 2.7% in patients between 43 and 54 years and 12.8% over 75 years [1, 2]. Later studies using optical coherence tomography (OCT) imaging for the detection of ERM found the prevalence to be higher (34.1%) [3].

For symptomatic ERM the treatment of choice is macular surgery. A pars-plana vitrectomy followed by the surgical removal of the epiretinal membrane is the standard procedure frequently performed since first description by Machemer in 1978 [4]. Intravitreally applied dyes are often used to facilitate the visualisation of the membrane. The surgical procedure generally leads to an improvement of visual function with a low complication rate [5, 6].

A seldom complication of an ERM removal is the recurrence of the ERM and the requirement of a re-operation. Several studies have assessed the recurrence rate and predictive factors [7–18].

Peeling of the ILM was shown to be associated with lower rates of ERM recurrence in comparison with ERM-only peeling [7–14]. However, the combined ERM and ILM removal has also been associated with increased morphological damage without improving functional outcomes [15, 16].

Lately, several studies have attempted to define OCT features that predispose for ERM recurrence. As such, the edge of a persistent ERM remnant, as well as post operative hyperreflective foci on the retinal surface have been found to be associated with ERM recurrence [17, 18].

The aim of this study is to analyse macular features by OCT in a large cohort of patients that underwent a macular surgery for idiopathic ERM and to find the incidence and associated factors of ERM persistence and recurrence.

## Methods

### Study design

This is a retrospective observational study, conducted at the Department of Ophthalmology of the Tübingen University Eye Hospital. The electronic medical charts of 920 eyes (878 patients) who underwent pars plana vitrectomy (ppV) and macula surgery for an ERM between February 2007 and May 2017 were retrospectively reviewed.

Eyes were excluded if the ERM was secondary to or associated with other disease affecting the macula, such as age-related macular degeneration, diabetic retinopathy, vascular occlusions, uveitis, high myopia of < −6dpt and previous intraocular surgery except for phacoemulsification. Eyes without follow-up OCT were also excluded. The number of patients that met the inclusion criteria was 645 (666 eyes).

ERM was diagnosed with fundoscopy and confirmed with OCT, as a highly reflective layer on the retinal surface. The OCT assessment was performed using a Spectralis OCT (Heidelberg Engineering, Heidelberg, Germany). In the majority of the eyes (88%) a 20° x 20° macula volume scan was available. In 12% only a radial cross scan and in less than 1% of eyes only horizontal and vertical cross scans through the fovea were performed. The analysed surgical characteristics included laterality, calibre of the cutter (20, 23 or 25 G), intraocular tamponade (balanced salt solution, air or gas), dye used for staining the vitreoretinal interface (Dual Blue, Brilliant Blue G, Trypan blue, Indocyanine Green or none), the presence or absence of a posterior vitreous detachment (PVD) as well as the surgeon.

Collected patient information were gender, age, duration of symptoms, baseline best corrected visual acuity (BCVA), status of the lens, intraoperative and postoperative complications, follow-up time and the requirement of a reoperation due to recurrence of ERM and final BCVA.

Three-port pars plana vitrectomy was performed utilising the Qube Pro vitrectomy surgical device from Ruck, Fritz Ruck Ophthalmologische Systeme GmbH, Germany. In cases where the posterior vitreous was still attached to the vitreo-retinal interface a PVD was induced by the surgeon. Following the removal of the posterior vitreous, peeling included the removal of all epimacular membranous structures with end-gripping forceps after the application of dyes. A post peeling staining was performed at the surgeon’s discretion.

**Fig. 1:**
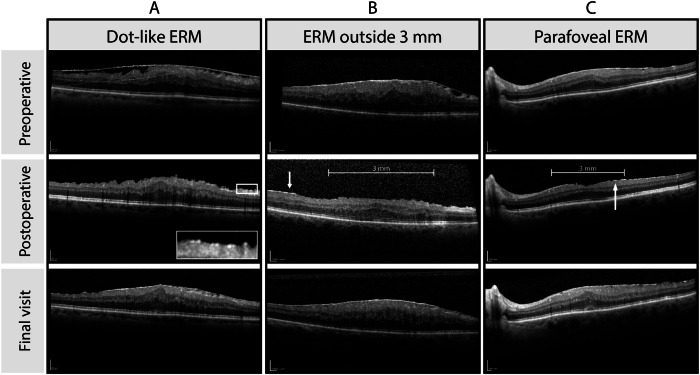
Types of postoperative ERM persistence on OCT after surgical ERM removal and subsequent formation of a new foveal ERM. **A** postoperative dot-like ERM, **B** postoperative large ( > 100 m) ERM persistence outside the parafoveal region, **C** postoperative large ERM persistence within the parafovea; Postoperative visit within 3 months after surgery; final visit is last available follow up.

The first postoperative OCT examination was performed within three months (on average (SD) 7.9 ( ± 4.6) weeks). The OCT examination at the final examination was the endpoint of the study. All examinations between the three months and the final visit were analysed additionally. OCT follow-up examination was performed using the built-in eye-tracking programme. Postoperative ERM was categorised in three groups; “no ERM”, “dot-like” ERM (length <100 µm) and “large ERM” (length ≥ 100 µm). In case of coexistence of dot-like and large ERM in one eye, it was classified as large ERM. The ERM location was categorised in three groups: foveal, parafoveal and outside the parafovea. The ERM was defined as foveal if it was identified within the area of 1.5 mm diameter centred on the fovea. The parafovea was defined as an annular area between the foveal circle and an outer circle of 3 mm diameter (Fig. 1).

The number of ERM fragments were counted and registered as more or less than three per OCT scan through the fovea. Central retinal thickness (CRT) was measured manually between the inner part of the retinal pigment epithelium and the retinal surface by using the built-in calliper of the OCT device. Presence or absence of intraretinal cystoid cavities was noted.

Any visible ERM detected by OCT examination within the three months after surgery was defined as persistent. Regression was defined in cases of persistent ERM that had disappeared at the last visit.

ERM recurrence was defined as reappearing ERM or growth of persistent ERM after the three months examination. Recurrence was analysed separately for each location (foveal, parafoveal and outside the 3 mm area).

For the statistical analysis JMP^®^, Version 15. (SAS Institute Inc., Cary, NC, 1989–2021) was used. Descriptive statistic was used for clinical and demographical data. Where appropriate, mean data is presented with Standard Deviation (SD) or 95% Confidence Interval (CI). To compare the groups with and without recurrence Two-sided T-test and Chi-square test were performed.

Odds ratios were calculated for associated risk factors. The most significant factors from the comparison of eyes *with* and without foveal recurrence were included into a logistic regression model. A *p* value of < 0.05 was considered statistically significant.

The study was approved by the institutional review board of the medical faculty of Tübingen University (Project No. 175/2020BO2) and was conducted in accordance with the principles of the Declaration of Helsinki.

## Results

### Clinical characteristics and demographics

The total of 920 eyes (886 patients) were treated with pars-plana vitrectomy and ERM removal between September 2006 and February 2018. Of these eyes, 666 (645 patients) met the inclusion criteria of the study. Mean age of the patients was 70.7 ( ± 7.3) years and 49.8% were female. The percentage of phakic eyes was 65.4%. Sixty-six eyes (9.9%) received a combined cataract and macular surgery (Table 1). Surgery was performed by 14 different surgeons, although 59% (393/666) of the eyes were operated by two surgeons. In 21% of cases a PVD was already present before surgery.

**Table 1:** Baseline characteristics of included eyes with Epiretinal membrane removal, *N* = 666 Eyes of 645 Patients.

Clinical Factors	Mean ± SD (Range) or No. (%) of Eyes
Age, years	70.7 ± 7.3 (range: 22–87)
Sex, female	331 (49.77)
Preoperative visual acuity, logMar	0.4 ± 0.2 (range: 0–1.4)
Pseudophakia	231 (34.68)
Combined cataract surgery	66 (9.91)
PVD before surgery visible in OCT	141 (21.17)
Vitrectomy gauge 20|23|25	13|650|2 (2.1|97.6|0.3)
Dye used DB|BB|TB|ICG| none	385|188|64|2|27 (57.8|18.9|9.6|0.3|4.1)
Material of endotamponade Air|Gas|BSS	128|31|509 (18.9|4.7|76.4)
Follow-up period, months	29.4 ± 28 (range: 1–129)

*SD* Standard deviation, *PVD* Posterior vitreous detachment, *OCT* Optical coherence tomography, *BB* Brilliant Blue G, *DB* Dual Blue, *ICG* Indocyanine Green, *TB* Trypan Blue, *BSS* Balanced salt saline.

Out of the 666 eyes with at least one follow-up OCT, 531 eyes underwent an examination within the first three months and 527 eyes an examination after the three months. The number of eyes with available OCT examinations within the postoperative three months and later was 392. The mean follow-up was 29.4 ± 27.9 months.

### Subgroup of patients with one follow-up examination

Within the three months examination ERM persistence was detected in 29.6% (157/531) and was found to be dot-like in 13.7% (73/531) and large in 15.8% (84/531). Foveal ERM persistence was noted in 1.9% (10/531; 9 large, 1 dot-like) of these eyes. Non-foveal ERM persistence was detected in 27.7% (147/531), was found to be parafoveal in 5.3% (28/531) and outside the central 3 mm diameter in 22.4% (119/531).

At the final visit (mean 29.4 months), a total of 39.8% (210/527) of the eyes presented either dot-like (6.1%, 32/527) or large (33.8%, 178/527) ERM. Foveal ERM was observed in 9.9% (52/527) and was large in all of these eyes. The overall percentage of any postoperative ERM was found to be significantly associated with the follow-up period in the logistic regression model (*p* = 0.03). When the last examination was within the first two years, ERM was found in 30.2% (145/458) of the eyes, within 2–5 years in 37% (50/134) and for 5–10 years in 50% (43/86) of the treated eyes. Reoperation for persistent or recurrent ERM was performed in 4.1% (27/666) of the eyes. The mean time period between the first surgery and reoperation was 19.2 months. The mean (range) duration between the onset of symptoms until surgery was 7.8 (1–36) months (*n* = 91).

### Subgroup of patients with ≥ two follow-up examinations

**Fig. 2:**
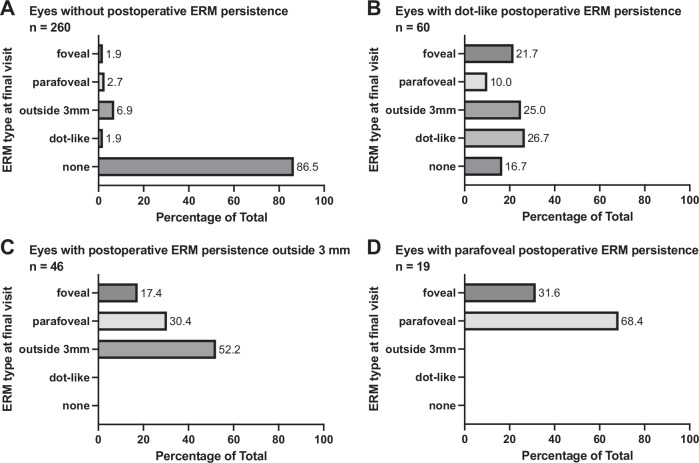
Development of Epiretinal membrane (ERM) type from postoperative visit within 3 months (ERM persistence) to the final visit. **A**–**D** show different types of ERM persistence. Dot-like = (ERM fragment size < 100 µm); foveal = (central edge within Ø 1.5 mm); parafoveal = (central edge between 1.5 mm Ø and 3 mm Ø); outside 3 mm = (central edge outside 3 mm Ø).

The subgroup of the 392 eyes with available OCT examinations within the postoperative three months and later, were analysed to determine the frequency of recurrence and the change of persistent ERM over time. Within this cohort, persistent ERM regardless of its location and size was noted in 33.7% (132/392). Growth of ERM was noted in 39% (51/132) of all eyes with persistent ERM. In 53% (70/132) the persistent ERM remained stable and in 7.6% (10/132; all dot-like ERM) a regression of ERM was noted during the observation period. The development of postoperative ERM persistence is shown in Fig. 2.

The overall recurrence rate was 21.9% (86/392) with 8.2% (32/392; all large) foveal recurrence, 3.3% (13/392) parafoveal and 10.5% (41/392) recurrence outside the 3 mm diameter.

In 84.4% (27/32) of the eyes with foveal ERM recurrence (6.9% of total), recurrence developed from growth of persistent ERM either from dot-like to large ERM (13/27), or by growth of large ERM with enlargement from non-foveal to foveal location (14/27). It is of note, that in only 15.6% (5/32) foveal ERM recurrence developed without a preceding ERM persistence.

Extrafoveal ERM recurrence was associated with progression from dot-like persistent ERM in 6.1% (24/392) and was new in 7.7% (30/392) (5 dot-like ERM, 25 large ERM).

The mean BCVA (mean LogMAR; SD) on the day before re-operation for ERM recurrence (0.45; 0.23) was significantly different from eyes without foveal ERM recurrence (0.25; 0.25), *p* = 0.015.

No statistically significant difference (mean; SD) was found for BCVA at the final visit between eyes with foveal ERM recurrence but without a re-operation (0.31; 0.32) compared to the cohort of eyes without foveal recurrence *p* = 0.269.

Table 2 summarises the clinical characteristics and the frequency of postoperative ERM persistence of all eyes with and without foveal ERM recurrence. Dot-like persistent ERM ( < 100 µm), and large ERM within and outside the parafovea were significantly associated with foveal ERM recurrence. No difference was found between the groups for age, sex, preoperative pseudophakia, preoperative BCVA, preoperative vitreous detachment, combined cataract surgery, use of dye, use of gas or air tamponade, final BCVA, intra-retinal cystoid cavities at any follow-up visit and surgeon’s experience. To analyse the effect of the surgeon’s experience, we grouped the two experienced surgeons that performed surgery in 393 out of all 666 eyes (each 193 and 200 respectively) and the group of 12 other surgeons that performed each between 1 and 55 surgeries. There was a small but not statistically significant difference in foveal ERM recurrence rate between the two experienced surgeons (9.3%) compared with the other 12 less experienced surgeons (10.8%, *p* = 0.37). We also investigated whether the preoperative grade of ERM was associated with an increased risk of persistence or recurrence post-surgery [19]. Preoperatively, the distribution of eyes across grade groups I-IV was 52 (13%), 102 (26%), 196 (50%), and 42 (11%), respectively. However, there was no statistically significant difference in the rate of postoperative ERM persistence among the grade groups (*n* = 132), which were 33%, 40%, 31%, and 31% (*p* = 0.44) for grades I-IV. Similarly, the rates of foveal ERM recurrence (*n* = 32) were 4%, 6%, 11%, and 7% (*p* = 0.28) across the same grade groups, indicating no significant association.

**Table 2:** Comparison of clinical characteristics between Eyes with and without foveal ERM recurrence.

Clinical characteristics	Without foveal ERM recurrence (*N* = 360)	With foveal ERM recurrence (*N* = 32)	*p* value
Age, years	70.8 ± 7.1	72.6 ± 10.8	^a^0.34
Sex, female	181 (50.3%)	11 (34.4%)	^b^0.09
Combined cataract surgery	37 (10.3%)	3 (9.4%)	^b^0.87
Preoperative visual acuity, logMar	0.45 ± 0.23	0.43 ± 0.20	^a^0.54
Performed by experienced surgeon*	244 (67.8%)	20 (62.5%)	^b^0.55
Preoperative pseudophakia	114 (31.7%)	13 (40.6%)	^b^0.30
Follow-up time, months	27.1 ± 26.3	27.1 ± 21.6	^a^1.00
Use of dye to stain membrane	12 (2.2%)	3 (9.4%)	^b^0.09
PVD before surgery	97 (26.9%)	10 (31.3%)	^b^0.60
Intraretinal cysts at any postoperative visit	116 (32.2%)	10 (31.3%)	^b^0.91
Gas/Air Tamponade versus BSS	91 (25.3%)	8 (25.0%)	^b^0.97
Postoperative “dot-like” ERM remnants <100 µm	51 (14.2%)	14 (43.8%)	^b^ < 0.001
Postoperative parafoveal ERM ≥ 100 µm	20 (5.6%)	6 (18.8%)	^b^0.004
Postoperative ERM ≥ 100 µm outside 3 mm diameter	38 (10.6%)	8 (25.0)%	^b^0.015

*ERM* Epiretinal membrane, *logMar* Logarithm of the minimum angle of resolution, *PVD* posterior vitreous detachment, *BSS* balanced salt saline.

^a^Two-sided T-test.

^b^ChiSquare-Test.

*”experienced” = senior surgeon that performed > mean number of ERM surgeries/surgeon.

The multifactorial logistic regression analysis, adjusted for significant or marginally significant factors in the comparison of eyes with and without foveal recurrence, confirmed a significant association between postoperative ERM persistence and foveal recurrence. Large ERM persistence within the parafovea showed the highest odds ratio for recurrence, followed by dot-like ERM persistence (any location) and large ERM persistence outside the area of 3 mm diameter (Table 3).

**Table 3:** Logistic regression analysis for foveal Epiretinal membrane recurrence.

Factors	Odds ratio (95% CI)	*p* value
Postoperative parafoveal ERM ≥ 100 µm	11.1 (3.3–37.2)	<0.001
Postoperative “dot-like” ERM remnants <100 µm	8.4 (3.4–20.8)	<0.001
Postoperative ERM ≥ 100 µm outside 3 mm diameter	5.8 (2.1–16)	<0.001
Sex, female	0.5 (0.2–1.2)	0.11
Use of dye to stain membrane	0.6 (0.1–2.4)	0.49

Although no use of dye was not an independent risk factor for recurrence in the multiple regression analysis, it was significantly associated with ERM persistence (OR 6.5 ;95% CI: 2.5–17; *p* < 0.001).

## Discussion

In the presented study, recurrence of the ERM after macular surgery was found in 21.9%. Foveal recurrence was seen in only 8.2% and was frequently associated with ERM persistence, which can be found in up to 29.6% of all eyes after macular surgery. Only 2.6% of eyes with foveal recurrence required a second ERM removal, which can be seen as the proportion of clinically significant ERM recurrence.

Previous studies have reported an ERM recurrence rate between 4 and 58% [8–12, 14, 17, 18, 20–27]. The high variability of these results is likely due to the methodological differences such as method of diagnosis, definitions of the endpoints, type of the surgical procedure and follow-up period.

Early studies are often based on biomicroscopic visibility of ERM recurrence [8, 24–26] and only in more recent studies, OCT imaging was primarily used for the detection of ERM enabling the detection of subtle recurrences that are invisible on fundus examination [9, 10, 12, 17, 18, 20, 27]. However, because consensus on a clear definition of recurrence is lacking, comparison between results from different studies is difficult [18].

The highest recurrence rates have been found after long follow-up periods and when non-foveal ERM was included [20, 27]. For example, after a follow up period of 5 years and including also extrafoveal ERM, a recurrence rate of up to 58% has been reported [20]. Of these 58%, 28% were found outside the fovea. This highlights the importance to include the location of recurrence into its definition. Another study that also explicitly included extrafoveal recurrence has reported a recurrence rate of 34% [27], which is still higher than the overall recurrence rate in our study population (21.9%). Beside the lower number of included eyes within these studies (*N* = 37 [20], *N* = 100 [27]), we suspect that another reason for the discrepancy could be that ERM recurrence was not well enough differentiated from ERM persistence, which was found in our population in over 29% of all eyes after ERM removal. Short-term postoperative OCT examination is however elementary to differentiate ERM persistence from late growth or new development of the ERM, namely recurrence.

The analysis of postoperative persistent ERM membranes seems even more important as there is growing evidence on their role for foveal recurrence. Few studies have specifically reported the clinical features of the ERM persistence and its role for recurrence [17, 18, 28]. In a cohort of 83 patients, a visible ERM remnant edge was associated with a significantly higher incidence of ERM recurrence during a mean follow-up period of 10 months. In this study, the recurrence rate, defined as regrowth of ERM covering the fovea, was as high as 42% in the group with ERM persistence versus 9,4% in the group without ERM persistence [17].

In line to our results, two other recent studies reported the ERM recurrence of 30.2% [28] and 39.5% [18] in association with early postoperative ERM membranes. In the study from 2021, a neighbouring remnant membrane, hyper-reflective dot(s) on the retinal surface and postoperative inner retinal wrinkling persisting were significantly associated with ERM recurrence [18]. In the study from 2023, any postoperative ERM fragments 3 days after surgery were associated with ERM recurrence [28].

Our study results confirm the association between postoperative ERM persistence and recurrence.

Furthermore, we have extended the analysis by differentiating between the location of ERM persistence (foveal, parafoveal, beyond) and its association with recurrence within and without the fovea.

We found that large persistent parafoveal ERM was most significantly associated with a foveal involvement at later follow-up visits. This finding underlines the role of the location of persistent ERM with respect to later foveal involvement and highlights the importance of the peeled area.

The dot-like persistent ERM was also associated with foveal regrowth of the ERM. Although the histological nature of these hyperreflective dots remains unknown, they could represent remnant fibrocellular tissue from which regrowth of ERM initiates. One could also suspect ILM remnants to be involved in the dot-like epiretinal hyperreflective lesions. On the other hand it has been reported that hyperreflective dots can also be seen in eyes after the ILM was peeled [18]. Different from larger ERM remnants, we have also noticed complete regression of the dot-like hyperreflective lesions, suggesting a temporary accumulation of cells or other materials. If the hyperreflective dots would indeed represent ILM remnants, a complete ILM peeling could potentially decrease the likelihood of the recurrence. The ILM peeling has been shown to be associated with lower recurrence rate [11, 13], but also has been associated with longer restoration time of macular morphology [29] and without significant influence on the postoperative vision and central macular thickness [14, 16].

The strength of this study is a clear definition of persistent and recurrent ERM as well as their size and location, the large number of patients, a long follow-up period and the strict exclusion of the eyes with coexisting morbidities. However, this study has also several limitations. Because of the retrospective design, the follow-up periods were not uniform and incidences possibly influenced by a selection bias. Furthermore, the lack of a standard OCT examination protocol as well as other imaging techniques like ‘en face’ and multicolour imaging [30] could possibly lead to an underestimation of the persistence and recurrence of the ERM. Our results are further limited by the size of the OCT scan that generally covered only the central 20° x 20°. Since double staining for ILM removal was on the discretion of the surgeons, its relevance for recurrence could not be analysed. OCT analysis was performed manually following strictly predefined categories by one single experienced physician to ensure consistency but with the downside of potential subjective influence on the study results. Due to the lack of a standard questionnaire only in 14% (91 eyes) the duration of symptoms was available. Although the analysis of the surgical parameters like use of different dyes, type of the endotamponade and the large number of the surgeons did not reveal statistically significant results they should also be seen as possible limitations of the study.

In summary, this study demonstrates that postoperative persistent ERM fragments are the most significant risk factors for later foveal ERM recurrence. The proximity of the persistent ERM to the fovea is associated with a higher foveal affection at later timepoints. Future large-scale studies addressing the role of double staining, the dimension of the peeled area or the use of the intraoperative OCT might be able to afford more insight into persistence and recurrence of ERM [29].

## Summary

### What was known before

- ERM recurrence is a seldom complication of ERM removal - Risk factors for ERM recurrence are not well understood.
- An association between postoperative ERM fragments and later ERM recurrence has been proposed but remains insufficiently explored.

### What this study adds

- Persistent ERM fragments found in the early postoperative period are a significant risk factor for recurrence.
- Fragments or the edge of the remnant membrane within an area of 3 mm diameter are the most important risk factor and the membrane removal should therefore exceed this area.

## Data Availability

The datasets generated and analysed during the current study are available from the corresponding author on reasonable request.
